# Cortical haemodynamic response during the verbal fluency task in patients with bipolar disorder and borderline personality disorder: a preliminary functional near-infrared spectroscopy study

**DOI:** 10.1186/s12888-021-03195-1

**Published:** 2021-04-20

**Authors:** Syeda Fabeha Husain, Tong-Boon Tang, Wilson W. Tam, Bach X. Tran, Cyrus S. Ho, Roger C. Ho

**Affiliations:** 1 Institute for Health Innovation and Technology (iHealthtech), National University of Singapore, Singapore, 117599 Singapore; 2 Department of Psychological Medicine, Yong Loo Lin School of Medicine, National University of Singapore, Singapore, 119228 Singapore; 3 Centre for Intelligent Signal and Imaging Research (CISIR), University Teknologi PETRONAS, Darul Ridzuan, 32610 Seri Iskandar, Perak Malaysia; 4 Alice Lee Centre for Nursing Studies, Yong Loo Lin School of Medicine, National University of Singapore, Singapore, 117597 Singapore; 5 Johns Hopkins Bloomberg School of Public Health, Johns Hopkins University, Baltimore, MD 21205 USA; 6 Institute for Preventive Medicine and Public Health, Hanoi Medical University, Hanoi, 116001 Vietnam; 7 Center of Excellence in Behavioral Medicine, Nguyen Tat Thanh University, Ho Chi Minh City, 70000 Vietnam

**Keywords:** Functional near-infrared spectroscopy, Verbal fluency task, Prefrontal cortex, Bipolar disorder, Borderline personality disorder

## Abstract

**Background:**

Functional near-infrared spectroscopy (fNIRS) is an emerging neuroimaging modality that provides a direct and quantitative assessment of cortical haemodynamic response during a cognitive task. It may be used to identify neurophysiological differences between psychiatric disorders with overlapping symptoms, such as bipolar disorder (BD) and borderline personality disorder (BPD). Hence, this preliminary study aimed to compare the cerebral haemodynamic function of healthy controls (HC), patients with BD and patients with BPD.

**Methods:**

Twenty-seven participants (9 HCs, 9 patients with BD and 9 patients with BPD) matched for age, gender, ethnicity and education were recruited. Relative oxy-haemoglobin and deoxy-haemoglobin changes in the frontotemporal cortex was monitored with a 52-channel fNIRS system during a verbal fluency task (VFT). VFT performance, clinical history and symptom severity were also noted.

**Results:**

Compared to HCs, both patient groups had lower mean oxy-haemoglobin in the frontotemporal cortex during the VFT. Moreover, mean oxy-haemoglobin in the left inferior frontal region is markedly lower in patients with BPD compared to patients with BD. Task performance, clinical history and symptom severity were not associated with mean oxy-haemoglobin levels.

**Conclusions:**

Prefrontal cortex activity is disrupted in patients with BD and BPD, but it is more extensive in BPD. These results provide further neurophysiological evidence for the separation of BPD from the bipolar spectrum. fNIRS could be a potential tool for assessing the frontal lobe function of patients who present with symptoms that are common to BD and BPD.

**Supplementary Information:**

The online version contains supplementary material available at 10.1186/s12888-021-03195-1.

## Background

Bipolar disorder (BD) is a chronic and severe mood disorder characterised by alternating episodes of mania, hypomania and depression. Borderline personality disorder (BPD) is another chronic and serious psychiatric disorder characterised by a pervasive pattern of unstable emotional regulation, interpersonal relationships, self-image and impulse control [1]. Despite this criteria, the boundary between BD and BPD is debatable, as it can be difficult to diagnose patients who present with both affective instability and impulsivity [2]. Across studies reporting the prevalence of these disorders, approximately 20% of patients receive a comorbid diagnosis [3–5]. Hence, it has been suggested that many BPD patients are better described as having BD, and that BPD should be considered as a variant of affective disorders [2]. Yet, majority of patients receive a diagnosis of BD or BPD alone, and comorbidity between BD and other personality disorders, and between BPD and mood or anxiety disorders are more common [3]. Moreover, misdiagnosis can be avoided with a detailed longitudinal history, to identify subtle differences in overlapping symptoms [2]. Mood changes in BD tend to be spontaneous, and shift from depression to elation, whereas mood changes in BPD are usually in response to environmental cues, and move from euthymia to anger [6]. Therefore, BD and BPD may not exist on a spectrum [6], and this hypothesis may be substantiated with objective and quantitative biomarkers, such as those obtained with neuroimaging techniques.

Neuroanatomical and neurophysiological abnormalities in BD and BPD are evident in neuroimaging data acquired using structural and functional magnetic resonance imaging (MRI), electroencephalography and positron emission tomography (PET). Compared to healthy controls (HC), both patient groups have smaller hippocampal volumes [7] and grey matter loss in the frontal and temporal lobes [8, 9]. Further grey matter loss occurs in the cerebellum, thalamus and putamen of patients with BD, while further grey matter loss in the amygdala and occipital lobe occurs in patients with BPD [8, 9]. Physiological abnormalities include altered connectivity within and between brain networks mediating social cognition, emotional regulation and self-referential processes [10], greater power in fast and slow oscillations [11] and glucose hypometabolism in the insula, brainstem, and frontal lobe [12]. Further hypometabolism occurs in the cerebellum of patients with BD, and in the hypothalamus, midbrain and striatum of patients with BPD [12]. In short, structural and functional abnormalities in BD and BPD relative to HCs overlap in some, but not all brain regions. When patient groups are compared, those with BPD have lower connectivity between the amygdala and prefrontal cortex [13], and lower uncorrected glucose metabolism in the prefrontal, insular and subgenual anterior cingulate cortex [12] than patients with BD. However, conclusions cannot be drawn, as direct comparisons between patients with BD and BPD are numbered [6]. Thus, studies with other imaging modalities, such as functional near-infrared spectroscopy (fNIRS), are needed.

Near-infrared light has the unique property of passing though tissues and being preferentially absorbed by haemoglobin [14]. As the absorbance spectra of haemoglobin is dependent on its binding with oxygen, fNIRS devices can continuously monitor both oxy-haemoglobin and deoxy-haemoglobin in the cerebral cortex [15]. fNIRS signals are a surrogate measure of the underlying neural activity, described by a phenomenon known as neurovascular coupling [16]. Regional neuron activity prompts an increase in blood flow and volume that is disproportionately higher than the metabolic demand of the brain. Therefore, a typical haemodynamic response involves a nett increase in oxy-haemoglobin, and a simultaneous slight decrease in deoxy-haemoglobin [17]. Since the changes in oxy-haemoglobin are greater than deoxy-haemoglobin, oxy-haemoglobin is used as marker of brain activity [18]. Although NIR light cannot reach subcortical regions, fNIRS is safe, non-invasive, non-restrictive, quiet, tolerant to motion and economical. These practical advantages make it a suitable tool for assessing psychiatric patients [19].

The verbal fluency task (VFT) is often conducted during fNIRS measurements as the conventional VFT is frequently used by clinicians to evaluate frontal lobe function in neuropsychiatric patients [20]. Various VFT paradigms and fNIRS signal processing methods have been published, but the protocol proposed by Takizawa et al. [21] was developed specifically for clinical settings. It has been extensively validated on psychiatric disorders, including BD [22–25] and BPD [26]. Yet, cortical haemodynamic response has not been directly compared between these two patient groups. Hence, the aim of this study was to compare fNIRS signals during the VFT between HCs, patients with BD and patients with BPD. We hypothesise that the mean oxy-haemoglobin changes in the frontotemporal cortex is the highest in HCs, followed by patients with BD and is the lowest in patients with BPD.

## Methods

### Participants

Twenty-seven participants (9 HCs, 9 patients with BD and 9 patients with BPD) who were between 21 and 50 years old were included in this study. All participants were female because these disorders are female predominant [1] and study participants were homogeneous by gender. Across the three diagnostic groups, subjects were matched for age, ethnicity and years of education. Depressive symptoms and psychosocial functioning for each participant were evaluated on the day of participation using the 17-item Hamilton rating scale for depression (HAM-D) [27] and global assessment of functioning (GAF) [28], respectively. In addition, manic symptoms in patients with BD and borderline personality traits in patients with BPD were assessed using the Young mania rating scale (YMRS) [29] and borderline personality questionnaire (BPQ), respectively [30]. As this was a preliminary study, YMRS scores of BPD patients and BPQ scores of BD patients were not obtained for all participants. Despite this limitation, manic and borderline personality traits were not predominant in BPD and BD, respectively.

HCs recruited from the community were assessed by a psychiatrist, and those included in this study were certified as being normal. They did not have a history of any psychiatric illnesses, including alcohol/substance abuse or addiction. HCs were excluded if they had conditions that could affect the central nervous system, including neurological illnesses such as epilepsy, traumatic brain injury, cerebrovascular diseases, respiratory diseases, hepatic diseases, kidney diseases, cancer or intellectual disability. Additionally, HCs who received psychotherapy in the past, had a HAM-D score of eight or higher on the day of participation [31], or reported drowsiness on the day of participation, were excluded.

Patients with BD or BPD were recruited from the outpatient psychiatric clinic at the National University Hospital, Singapore. They were diagnosed by a psychiatrist, according to the criteria in the fifth edition of the Diagnostic and Statistical Manual of Mental Disorders (DSM-5) for BD or BPD [1], using the Structured Clinical Interview for the DMS-5 [32]. Patients were excluded if they had any neurological illnesses, traumatic brain injury, cerebrovascular diseases, respiratory diseases, hepatic diseases, kidney diseases, cancer, intellectual disability or alcohol/substance abuse or addiction. In addition, those who received psychotherapy in the past, or reported drowsiness on the day of participation, were excluded.

Study details were fully explained to participants, and their written informed consent was obtained. *The authors assert that all procedures contributing to this work comply with the ethical standards of* the Declaration of Helsinki, and the ethical principles in the Belmont Report. It was approved by the Domain Specific Review Board of the National Healthcare Group, Singapore (protocol number 2017/00509).

### Verbal fluency task

Before fNIRS measurements were taken, participants watched a demonstration video, in which they were asked to remain seated, avoid excessive body or head movements, and focus on a cross displayed during the VFT. The VFT paradigm (Supplementary Fig. 1) consisted of a 30s pre-task period, 60s task period, and a 70s post-task period. Participants were asked to say “A, B, C, D, E” aloud and repeatedly during the pre- and post-task periods. During the task period, they were instructed to generate as many words as possible, beginning with A, F and S for 20 s per letter. The total number of unique words, enunciated within the task period, was recorded as the task performance. Before the actual trial, participants were asked to practice the VFT for a shorter duration, and with the letters H, B and P. This ensured all participants understood the task and responded to the cues correctly during the actual trial.

### NIRS measurement

A 52-channel fNIRS system (ETG-4000. Hitachi Medical Co., Tokyo, Japan) with 2 NIR light wavelengths (695 and 830 nm) was used to measure relative oxy-haemoglobin and deoxy-haemoglobin changes [33]. Emitter and detector optodes were arranged 3 cm apart, and the area between each emitter and detector pair is called a channel. Anatomically, channels locations are cortical regions 2–3 cm beneath the skin and scalp surface [34]. Optodes were placed on the forehead and scalp, with the lowest optodes being along the T4-Fpz-T3 line of the 10/20 system. This arrangement allowed for haemodynamic response in the bilateral prefrontal cortex, frontopolar cortex, and the anterior regions of the superior and middle temporal cortices to be measured. These approximate channel locations are based on the anatomical craniocerebral correction of the international 10/20 system.

### fNIRS signal analysis

**Fig. 1:**
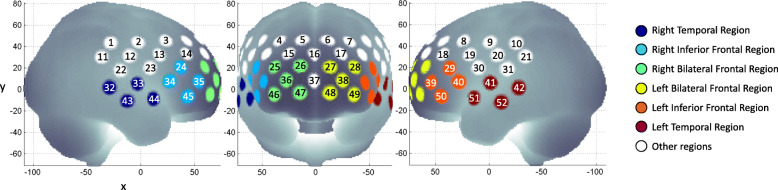
Cortical regions of interest. Coloured channels were divided into 6 ROIs

fNIRS signals were processed according to the method described by Takizawa et al. [21]. The modified Beer-Lambert law was used to derive relative changes in oxy-haemoglobin, deoxy-haemoglobin and total haemoglobin from optical densities. Haemoglobin changes during the task period were normalised by linear fitting between a 10 s baseline at the end of the pre-task period, and a 5 s post-task baseline period that is 50 s into the post-task period (Supplementary Fig. 1). Short term motion artefacts were removed using a moving average factor of 5. An algorithm identifying channels with body movement artefacts, or high and low frequency noise was applied. Artefact channels were identified and removed from further analysis using an algorithm for body movement artefacts, or high and low frequency noise. The mean oxy-haemoglobin and deoxy-haemoglobin changes during the pre-task and task periods at each region of interest (ROI; Fig. 1) was determined for each subject. These ROIs were first proposed by Chou et al. [35] and are defined as the right temporal region (channels 32, 33, 43 and 44), right inferior frontal region (channels 24, 34, 35 and 45), right bilateral frontal region (channels 25, 26, 36, 46 and 47), left bilateral frontal region (channels 27, 28, 38, 48 and 49), left inferior frontal region (channels 29, 39, 40 and 50) and left temporal region (channels 41, 42, 51 and 52). Remaining channels were excluded from the analysis (channels 1–23, 30, 31 and 37). Channel positions were plotted using NFRI functions toolbox [36]. Since changes in oxy-haemoglobin are larger than deoxy-haemoglobin [18], results for the latter are reported in supplementary materials.

### Statistical analysis

To determine if activation during the VFT occurred for each diagnostic group at each ROI, Student’s paired t-test was used to compare mean oxy-haemoglobin during the pre-task baseline period and task period. The effect of diagnostic group on categorical variables was determined using chi-square test, while Student’s t-test or one-way analysis of variance (ANOVA) with Bonferroni corrected post-hoc pairwise comparisons, was used to determine the effect of diagnostic group on continuous variables. Categorical variables were gender, ethnicity, handedness, family psychiatric history, past admission to psychiatric ward and treatment with psychotropic drugs. Psychotropic drugs were further classified into antidepressants, anxiolytics and sedatives, antipsychotics and mood stabilisers (Supplementary Table 1). Continuous variables were age, years of education, number of words generated, mean oxy-haemoglobin and mean deoxy-haemoglobin at each ROI, GAF score, HAM-D score, YMRS score, BPQ score, age at psychiatric illness onset, duration of psychiatric illness and equivalent doses of antidepressants, anxiolytics and sedatives, as well as antipsychotics. Equivalent doses were calculated based on published mean dose ratios. Reference drugs were fluoxetine, diazepam and chlorpromazine, for antidepressants, anxiolytics and sedatives, and antipsychotics, respectively [37, 38]. The combined equivalent dose was calculated for patients receiving more than one drug in each class.

Subsequent regression analysis was carried out when mean oxy-haemoglobin at any ROI differed between patient groups. Mean oxy-haemoglobin was the dependent variable, while independent variables included in the model were diagnosis and any other demographic, behavioural or clinical variables that differed between patient groups. Additionally, associations between mean oxy-haemoglobin at these ROIs with behavioural or clinical variables was determined using Pearson’s correlation or Student’s t-test.

All tests were two-tailed, with a significance level of *p* ≤ 0.05. Data are expressed as mean and standard deviation. Statistical analysis was done on SPSS Statistic 21.0 (IBM).

## Results

### Sample characteristics

The three diagnostic groups did not differ in any demographic, behavioural or clinical variables, except GAF and HAM-D scores (Table 1). Unsurprisingly, HCs had higher GAF scores (*F* = 21, *p* ≤ 0.001) and lower HAM-D scores (*F* = 16.7, *p* ≤ 0.001) than patients with BD [GAF: *p* ≤ 0.001, 95% CI, (9.5 to 35); HAM-D: *p* = 0.006, 95% CI, (2.2 to 14.5)] and patients with BPD [GAF: *p* ≤ 0.001, 95% CI, (18.4 to 43.9); HAM-D: *p* ≤ 0.001, 95% CI, (7.3 to 19.2)], but patient groups did not differ in GAF or HAM-D scores. When medication status was compared, a larger proportion of patients with BPD were receiving antidepressants than patients with BD [*X*^2^(2, *n* = 18) = 6.9, *p* = 0.029].

**Table 1:** Demographic and clinical data (*N* = 27)

	HC	BD	BPD	***p***-value
Age (years)	33.4 ± 10.2	34.6 ± 10.4	34 ± 9.9	0.974
Ethnicity				1.00
Chinese	8 (88.9%)	8 (88.9%)	8 (88.9%)	
Indian	1 (11.1%)	1 (11.1%)	1 (11.1%)	
Education (years)	16 ± 1.5	16 ± 2.1	14 ± 2.1	0.059
Handedness ^a^				0.258
Right	9 (100%)	5 (71.4%)	8 (100%)	
Left	0	1 (14.3%)	0	
Ambidextrous	0	1 (14.3%)	0	
Number of words ^a^	16.8 ± 4.1	15.4 ± 4.3	16 ± 5	0.813
Family history of psychiatric illness ^a^	2 (25%)	4 (50%)	4 (44.4%)	0.561
Age at illness onset (years)	–	25.3 ± 8.1	23.9 ± 9.3	0.729
Duration of illness (years)	–	9.2 ± 8.1	10.1 ± 6.6	0.802
Past admission into psychiatric ward	–	7 (77.8%)	8 (88.9%)	0.500
GAF score ^b^	93.3 ± 8.7	71.1 ± 12.7	62.2 ± 9.7	**≤0.001**
HAM-D score ^a, b^	2.9 ± 2.5	11.3 ± 6.3	16.1 ± 5.3	**≤0.001**
YMRS score ^a^	–	2.1 ± 3.4	–	–
BPQ score	–	31 ± 22.7	57.7 ± 5.1	0.176
Pharmacotherapy	–	9 (100%)	9 (100%)	–
Antidepressants	–	4 (44.4%)	9 (100%)	**0.029**
Anxiolytics & sedatives	–	4 (44.4%)	2 (22.2%)	0.310
Antipsychotics	–	3 (33.3%)	2 (22.2%)	0.500
Mood stabilisers	–	6 (66.7%)	5 (55.6%)	0.500
Fluoxetine eq. dose (mg/day)	–	44 ± 30.2	29.3 ± 17.3	0.282
Diazepam eq. dose (mg/day)	–	12.1 ± 9.7	7.1 ± 4.1	0.540
Chlorpromazine eq. dose (mg/day)	–	244 ± 139.3	303 ± 214.3	0.726

Mean ± SD are shown and *p*-values ≤0.05 are in bold.

^a^ Complete demographic and clinical data were not obtained for all subjects (Known handedness in healthy controls, *n* = 9; patients with bipolar disorder, *n* = 7; patients with borderline personality disorder, *n* = 8. Known number of words in healthy controls, *n* = 9; patients with bipolar disorder, *n* = 8; patients with borderline personality disorder, *n* = 9. Known family history of psychiatric disorder in healthy controls, *n* = 8; patients with bipolar disorder, *n* = 9; patients with borderline personality disorder, *n* = 9. Known HAM-D score in healthy controls, *n* = 9; patients with bipolar disorder, *n* = 8; patients with borderline personality disorder, *n* = 9. Known YMRS score in patients with bipolar disorder, *n* = 7. Known BPQ score in patients with bipolar disorder, *n* = 3; patients with borderline personality disorder, *n* = 9.)

^b^ Post-hoc test showed statistically significant differences in GAF and HAM-D scores between healthy controls and patients with bipolar disorder (GAF, *p* ≤ 0.001; HAM-D, *p* = 0.006) and between healthy controls and patients with borderline personality disorder (*p* ≤ 0.001), but not between patient groups (GAF, *p* = 0.255; HAM-D, *p* = 0.159)

### Haemodynamic response during the verbal fluency task

**Fig. 2:**
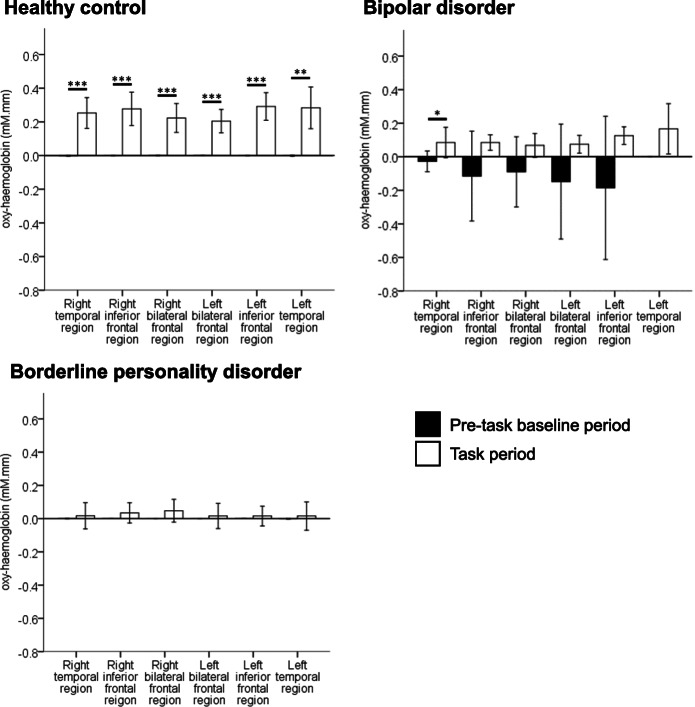
Cortical areas of activation. Mean oxy-haemoglobin between the pre-task baseline period and the task period was compared for each diagnostic group per ROI using paired t-test (*p ≤ 0.05, **p ≤ 0.01, ***p ≤ 0.001). Data are presented as mean ± SD

The mean oxy-haemoglobin during the task period was higher than the mean oxy-haemoglobin during the pre-task baseline period in all ROIs for HCs [Fig. 2; right temporal region: *t* = − 6.6, *p* ≤ 0.001, 95% CI, (− 0.3 to − 0.2); right inferior frontal region: *t* = − 6.4, *p* ≤ 0.001, 95% CI, (− 0.4 to − 0.1); right bilateral frontal region: *t* = − 6, *p* ≤ 0.001, 95% CI, (− 0.3 to − 0.1); left bilateral frontal region: *t* = − 6.8, *p* ≤ 0.001, 95% CI, (− 0.3 to − 0.1); left inferior frontal region: *t* = − 8.2, *p* ≤ 0.001, 95% CI, (− 0.4 to − 0.2); left temporal region: *t* = − 6.4, *p* = 0.003, 95% CI, (− 0.4 to − 0.2)]. For each patient group, there were no statistically significant differences in mean oxy-haemoglobin between the pre-task baseline period and the task period in all ROIs, except in the right temporal region for patients with BD [*t* = − 2.4, *p* = 0.047, 95% CI, (− 0.2 to 0)]. In HCs, mean deoxy-haemoglobin during the pre-task baseline period was higher than the mean deoxy-haemoglobin during the task period (Supplementary Fig. 2) in the right bilateral frontal [*t* = 4, *p* = 0.004, 95% CI, (0 to 0.1)] and the left bilateral frontal regions only [*t* = 5.5, *p* = 0.001, 95% CI, (0 to 0.1)]. There were no statistically significant differences in mean deoxy-haemoglobin between the pre-task baseline period and the task period in all ROIs for patient with BD and patients with BPD. This suggests that activation may occur in the frontotemporal cortex in HCs during the VFT, but not in patients with BD and patients with BPD.

**Fig. 3:**
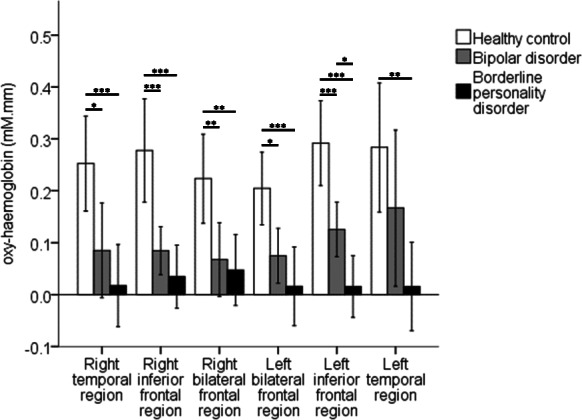
Comparison of mean oxy-haemoglobin during the task period. One-way ANOVA with Bonferroni corrected post-hoc pairwise t-tests were used to compare mean oxy-haemoglobin during the task period between diagnostic groups per ROI (*p ≤ 0.05, **p ≤ 0.01, ***p ≤ 0.001). Data are presented as mean ± SD

Compared to HCs, patient groups had lower mean oxy-haemoglobin during the task period in all ROIs (Fig. 3; right temporal region: *F* = 10.7, *p* = 0.001; right inferior frontal region: *F* = 16.6, *p* ≤ 0.001; right bilateral frontal region: *F* = 8.6, *p* = 0.001; left bilateral frontal region: *F* = 11, *p* ≤ 0.001; left inferior frontal region: *F* = 23.8, *p* ≤ 0.001; left temporal region: *F* = 7.2, *p* = 0.005). Specifically, patients with BD have lower mean oxy-haemoglobin than HCs during the task period in all ROIs, except the left temporal region [Right temporal region: *p* = 0.015, 95% CI, (0 to 0.3); right inferior frontal region: *p* = 0.001, 95% CI, (0.1 to 0.3), right bilateral frontal region: *p* = 0.008, 95% CI, (0 to 0.3), left bilateral frontal region: *p* = 0.013, 95% CI, (0 to 0.2); left inferior frontal region: *p* = 0.001, 95% CI, (− 0.3 to − 0.1)]. Similarly, patients with BPD have lower mean oxy-haemoglobin that HCs during the task period in all ROIs [Right temporal region: *p* = 0.001, 95% CI, (0.1 to 0.3); right inferior frontal region: *p* ≤ 0.001, 95% CI, (0.1 to 0.4); right bilateral frontal region: *p* = 0.003, 95% CI, (0.1 to 0.3); left bilateral frontal region: *p* ≤ 0.001, 95% CI, (0.1 to 0.3); left inferior frontal region: *p* ≤ 0.001, 95% CI, (0.2 to 0.4); left temporal region: *p* = 0.005, 95% CI, (0.1 to 0.5)]. Mean deoxy-haemoglobin during the task periods only differed between diagnostic groups (Supplementary Fig. 3) in the right bilateral frontal region (*F* = 6.8, *p* = 0.005) and the left bilateral frontal region (*F* = 5.6, *p* = 0.010). Specifically, HCs had lower mean deoxy-haemoglobin than patients with BPD during the task period in these ROIs [right bilateral frontal region: *p* = 0.004, 95% CI, (0 to 0.1); left bilateral frontal region: *p* = 0.010, 95% CI, (0 to 0.1)]. These results suggest that patient groups may have reduced haemodynamic response in the frontotemporal cortex.

**Fig. 4:**
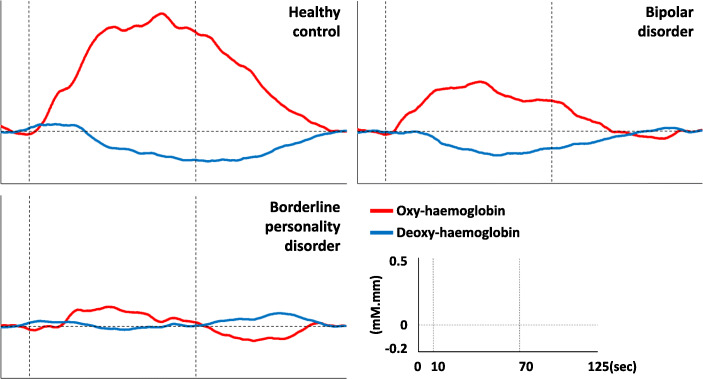
Average oxy-haemoglobin and deoxy-haemoglobin waveforms at the left inferior frontal region. Dotted vertical lines demarcate the start and end of the VFT task period

When mean oxy-haemoglobin during the task period at each ROI are compared between patient groups (Fig. 3), patients with BPD have lower mean oxyhaemoglobin than patients with BD in the left inferior frontal region only [*p* = 0.035, 95% CI, (0 to 0.2)]. Subsequent linear regression (Adjusted R^2^ = 0.3117) showed that diagnosis (*β* = − 0.099, S.E. = 0.045, *p* = 0.045), but not antidepressant status (*β* = − 0.021, S.E. = 0.050, *p* = 0.688), is associated with mean oxy-haemoglobin in the left inferior frontal region during the task period. Mean deoxy-haemoglobin during the task period did not differ between patient groups in all ROIs (Supplementary Fig. 3, *p* > 0.05). Nevertheless, average waveforms show a greater decline in deoxy-haemoglobin during the task period in patients with BD than patients with BPD (Fig. 4). In addition, mean oxy-haemoglobin at the left inferior frontal region was not associated with any behavioural or clinical variables amongst patients (Supplementary Table 2). Taken together, the results suggest that haemodynamic dysfunction in the left inferior frontal cortex may be more extensive in patients with BPD than patients with BD.

## Discussion

The present study lends further support for the distinction of BD and BPD in psychiatric nosology. Similar to earlier fNIRS reports [22–26], reduced haemodynamic response occurred primarily in the frontal and temporal cortex of both patient groups in our sample. Likewise, MRI and PET of the frontotemporal regions in patients with BD and BPD revealed grey matter loss and reduced glucose metabolism in other studies [8, 9, 12]. This suggests that frontotemporal oxy-haemoglobin measured by fNIRS may be a potential biomarker for both BD and BPD. Interestingly, Ghaemi et al. [39] described BD as a biological disease based on genetics, but BPD as a psychologically caused clinical picture. However, a genome-wide association study by Witt et al. [40] identified genetic overlaps between BD and BPD. Furthermore, epigenetic modifications of serotonin 3A receptor genes occur in both BD and BPD patients with childhood maltreatment [41]. The conventional treatment strategies are mood-stabilisers for patients with BD, and psychotherapy for patients with BPD [6]. Yet, patients with BD may benefit from integrated psychotherapeutic techniques [42], while patients with BPD may respond to antidepressants, second-generation antipsychotics and mood stabilisers [43]. Therefore, neuroimaging, genetic and pharmacological approaches provide evidence for the neurobiological basis of both BD and BPD.

The current approach for the differential diagnosis of BD and BPD relies on particular symptoms in the existing clinical criteria [44]. Symptoms such as reduced need for sleep [45], elevated mood, increased goal-directed activities and episodicity [46] are more closely associated with BD, while abandonment fears, identity disturbance [47], attempted suicide and childhood trauma [45] are more closely associated with BPD. Questionnaires may aid in distinguishing BD and BPD by assessing these symptoms. These include the HAM-D, the Hamilton anxiety rating scale, the YMRS, the borderline personality disorder severity index-IV [48] and the Personality Inventory for DSM-5 [49]. Still, symptoms alone are inadequate diagnostic validators, and the diagnostic criteria needs to be supported by biomarkers, including functional neuroimaging markers [39]. In addition to advancing our knowledge of neurobiology, neuroimaging technologies may potentially be translated to aid in the differential diagnosis of BD and BPD [45].

A notable finding in this study is that haemodynamic dysfunction in the left inferior frontal region is more extensive in patients with BPD than patients with BD. To our knowledge, this is the first study to report differences in prefrontal cortex activity during a cognitive task between these patient groups. This is in line with earlier reports of lower prefrontal cortex glucose metabolism [12] and lower connectivity between the prefrontal cortex and amygdala [13] in BPD compared to BD. The lower prefrontal cortex activity in patients with BPD may be linked to poorer response inhibition, planning [50], decision-making [51] and psychosocial functioning [52] in these patients compared to those with BD. Furthermore, frontotemporal oxy-haemoglobin changes measured with the fNIRS protocol used in this study has been previously shown to distinguish patients with major depression from both HCs and patients with BD or schizophrenia [53]. Thus, haemodynamic response in the left inferior frontal region during the VFT may be a potential biomarker to differentiate BPD from BD in clinical settings. Larger samples are needed to validate this hypothesis, before the fNIRS-VFT paradigm may be used as a supplementary test to support a diagnosis.

This study has several limitations, beginning with the small sample size. Consequently, cortical activity of disorder subtypes could not be compared, such as BD or BPD with and without current depressive symptoms, and BD with and without borderline personality traits. Thus, replicate studies on larger samples of patients with BD or BPD are needed. Secondly, adults were recruited in this study, whereas the onset of BD and BPD usually occurs in young adulthood and adolescence, respectively [45]. fNIRS signals during the VFT in adolescents with BD has been reported elsewhere [54], and a direct comparison of haemodynamic response between adolescents with BD and BPD may enhance our knowledge of their aetiology. Moreover, a prospective study on adolescents may establish a causal relationship between disorder onset and haemodynamic dysfunction, which could not be established in this cross-sectional study. Thirdly, pharmacotherapy varied greatly between patients and the effect of each drug on haemodynamic response is not clear. Still, previous studies on larger samples of psychiatric patients with heterogeneous pharmacotherapy suggest the effect of such medication on fNIRS signals is minimal [55]. Though beyond the scope of this study, future fNIRS studies comparing male patients with BD and BPD may be of interest.

## Conclusions

Findings from this study provide preliminary evidence for future research on functional neuroimaging biomarkers for the differential diagnosis of BD and BPD. Haemodynamic response in the left inferior frontal cortex differed between healthy individuals, patients with BD and patients with BPD. Given that fNIRS signals are a direct and objective measure of neurophysiology, these observations support the separation of BPD from the bipolar spectrum.

[Main text word count: 3489].

## Supplementary Information

**Additional file 1.** Cortical haemodynamic response during the verbal fluency task in patients with bipolar disorder and borderline personality disorder: a preliminary functional near-infrared spectroscopy study.

## Data Availability

The datasets used and/or analysed during the current study are available from the corresponding author on reasonable request.

## Abbreviations

BD: Bipolar disorder
BPD: Borderline personality disorder
BPQ: Borderline personality questionnaire
DSM-5: Diagnostic and Statistical Manual of Mental Disorders
fNIRS: Functional near-infrared spectroscopy
GAF: Global assessment of functioning
HAM-D: Hamilton rating scale for depression
HC: Healthy control
MRI: Magnetic resonance imaging
PET: Positron emission tomography
ROI: Region of interest
VFT: Verbal fluency task
YMRS: Young mania rating scale
