# Supplementary material for: Cortical haemodynamic response during the verbal fluency task in patients with bipolar disorder and borderline personality disorder: a preliminary functional near-infrared spectroscopy study
Source: BMC Psychiatry. 2021 Apr 20;21:201. doi: 10.1186/s12888-021-03195-1 (PMC8056702; doi:10.1186/s12888-021-03195-1)
Supplement: Supplementary file 1 — Additional file 1. Cortical haemodynamic response during the verbal fluency task in patients with bipolar disorder and borderline personality disorder: a preliminary functional near-infrared spectroscopy study. [file 12888_2021_3195_MOESM1_ESM.docx]

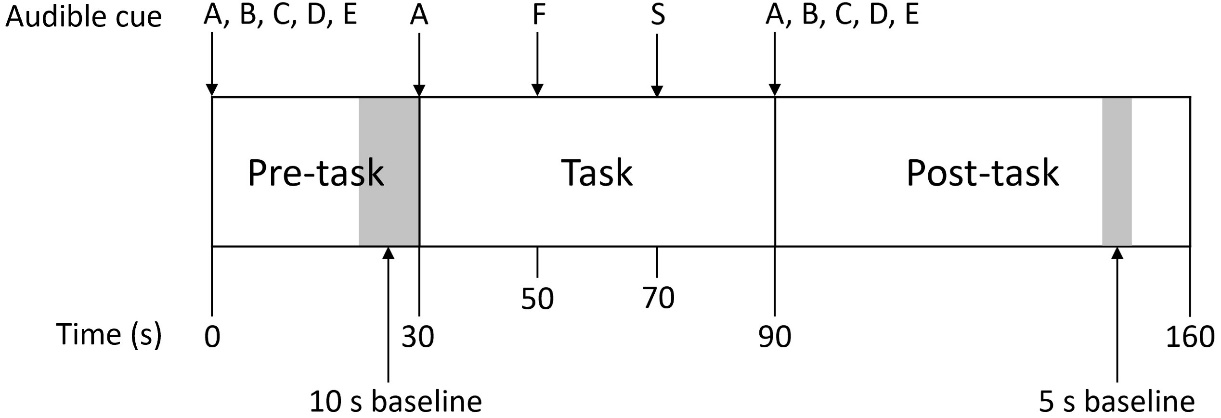


**Supplementary Figure 1.** The verbal fluency task protocol.

**Suplementary table 1. Medication details.**

|  | **Bipolar disorder** | | **Borderline personality disorder** | |
| --- | --- | --- | --- | --- |
|  | n | Dose (mg/day) | n | Dose (mg/day) |
| Antidepressants |  |  |  |  |
| *Selective serotonin reuptake inhibitors* |  |  |  |  |
| Escitalopram | 0 | - | 1 | 20 |
| Fluoxetine | 1 | 20 | 3 | 26.7 ± 11.5 |
| Fluvoxamine | 1 | 100 | 3 | 116.7 ± 76.4 |
| Paroxetine | 0 | - | 1 | 12.5 |
| Sertraline | 1 | 100 | 0 | - |
| *Serotonin and norepinephrine reuptake inhibitors* |  |  |  |  |
| Duloxetine | 1 | 60 | 0 | - |
| *Noradrenergic and specific serotonergic antidepressant* |  |  |  |  |
| Mirtazapine | 0 | - | 1 | 15 |
| *Other antidepressants* |  |  |  |  |
| Agomelatine | 0 | - | 1 | 25 |
| *Combination antidepressants* |  |  |  |  |
| Mirtazapine & escitalopram | 0 | - | 1 | - |
| Anxiolytics and sedatives |  |  |  |  |
| Alprazolam | 1 | 1 | 0 | - |
| Diazepam | 2 | 7.5 ± 3.5 | 0 | - |
| Clonazepam | 1 | 1 | 0 | - |
| Lorazepam | 1 | 0.5 | 1 | 1 |
| Zopiclone | 1 | 7.5 | 1 | 15 |
| *Combination anxiolytics and sedatives* |  |  |  |  |
| Alprazolam & clonazepam | 1 | - | 0 | - |
| Diazepam & lorazepam | 1 | - | 0 | - |
| Antipsychotics |  |  |  |  |
| Asenapine | 1 | 10 | 0 | - |
| Quetiapine | 1 | 200 | 2 | 200 ± 141.4 |
| Risperidone | 1 | 2 | 0 | - |
| Mood stabilisers |  |  |  |  |
| Lamotrigine | 1 | 100 | 1 | 150 |
| Lithium carbonate | 2 | 600 ± 282.8 | 0 | - |
| Sodium valproate | 5 | 660 ± 320.9 | 3 | 533.3 ± 251.7 |
| Topiramate | 0 | - | 1 | 50 |
| *Combination mood stabilisers* |  |  |  |  |
| Lamotrigine & sodium valproate | 1 | - | 0 | - |
| Lithium carbonate & sodium valproate | 1 | - | 0 | - |


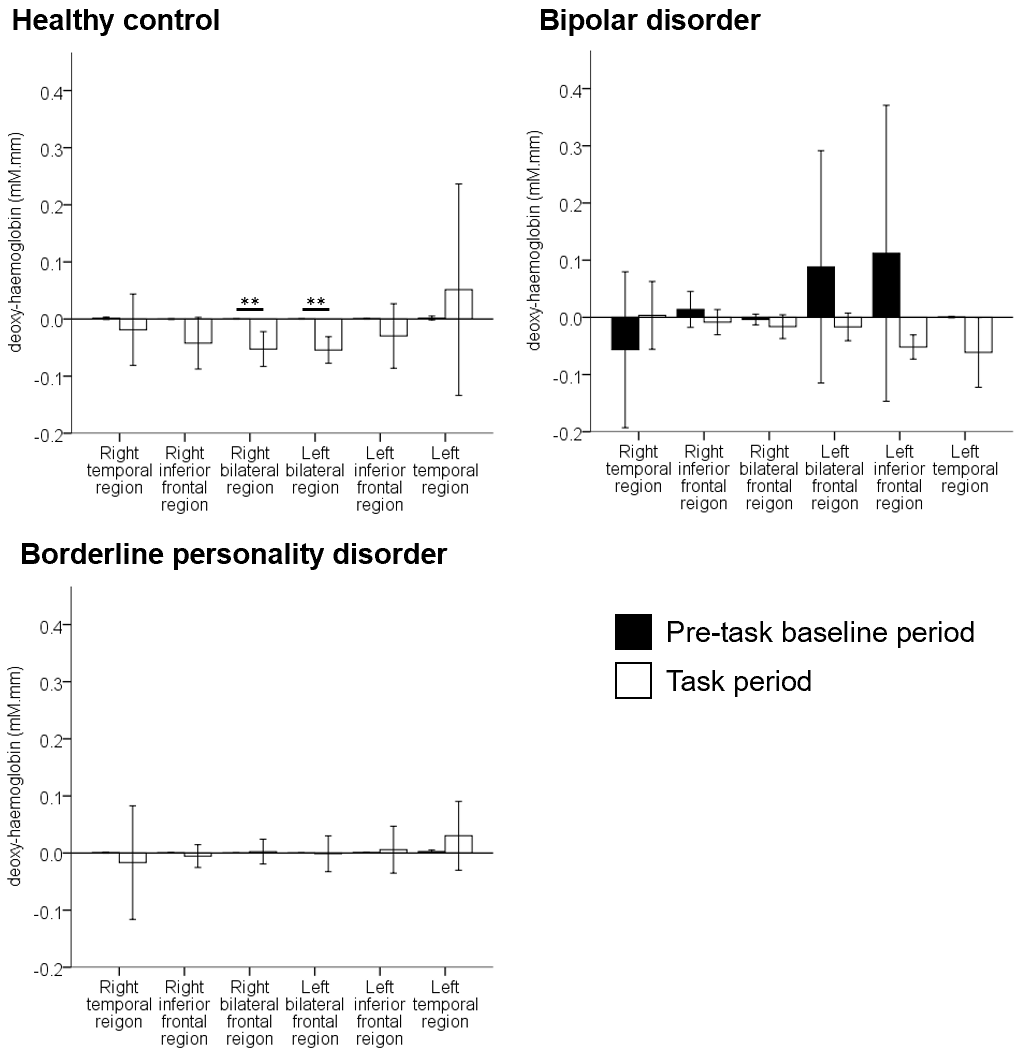


**Supplementary figure 2. Changes in deoxy-haemoglobin between periods.** Mean deoxy-haemoglobin between the pre-task baseline period and the task period was compared for each diagnostic group per ROI using paired t-test (***p*≤0.01). Data are presented as mean ± SD.


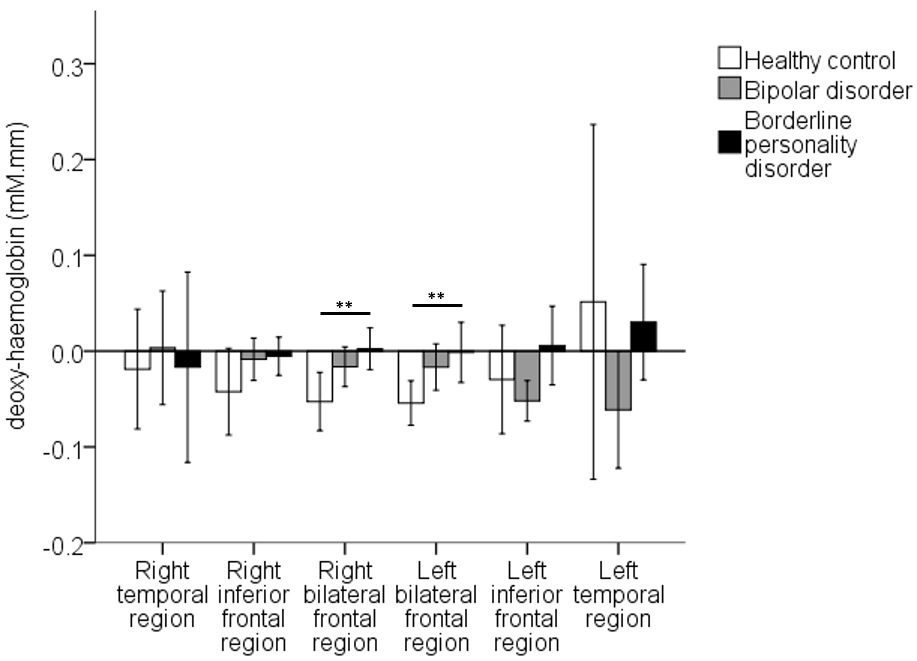


**Supplementary figure 3. Comparison of mean deoxy-haemoglobin during the task period.** One-way ANOVA with Bonferroni corrected post-hoc pairwise t-tests were used to compare mean deoxy-haemoglobin during the task period between diagnostic groups per ROI (***p*≤0.01). Data are presented as mean ± SD.

**Supplementary table 2.** Associations between mean oxy-haemoglobin at the left inferior frontal region and behavioural or clinical variables for patients with BD and BPD.

|  | Test statistic | *p*-value |
| --- | --- | --- |
| Number of words ^a^ | -0.029 | 0.911 |
| Family psychiatric history ^b^ | 0.679 | 0.508 |
| GAF score ^a^ | 0.183 | 0.467 |
| HAM-D score ^a^ | -0.241 | 0.352 |
| YMRS score ^a^ | 0.332 | 0.466 |
| BPQ score ^a^ | 0.358 | 0.344 |
| Age at illness onset (years) ^a^ | 0.005 | 0.984 |
| Duration of illness (years) ^a^ | -0.102 | 0.687 |
| Past admission to psychiatric ward ^b^ | -0.373 | 0.714 |
| Fluoxetine eq. dose (mg/day) ^a^ | -0.117 | 0.703 |
| Diazepam eq. (mg/day) ^a^ | 0.017 | 0.975 |
| Chlorpromazine eq. dose (mg/day) ^a^ | -0.524 | 0.365 |
| Mood stabiliser ^b^ | -0.246 | 0.809 |

^a^ Pearson’s correlation test.

^b^ Student’s t-test.
